# Advances in Theranostic Approaches and Emerging Biomarkers of Diabetes Mellitus

**DOI:** 10.3390/diagnostics14192207

**Published:** 2024-10-03

**Authors:** Gulali Aktas

**Affiliations:** Internal Medicine Department, Abant Izzet Baysal University Hospital, 14030 Bolu, Turkey; draliaktas@yahoo.com

Type 2 diabetes mellitus is one of the most common chronic conditions in the world. It is associated with significant morbidity, mortality, and healthcare costs. Early diagnosis of type 2 diabetes mellitus and its complications is vital for reducing or preventing morbidity and mortality of diabetes mellitus and related conditions. Recent studies reported novel biomarkers of diabetes mellitus and chronic diabetic complications. Moreover, theranostic approaches have been developed in the diagnosis and treatment of diabetes mellitus.

Theranostics refers to using a radioactive agent to establish a diagnosis and another radioactive drug to deliver therapy to treat cancer. On the other hand, theranostic approaches are also being studied in type 2 diabetes mellitus and diabetic complications. Nanomedicines are another type of theranostics in the diagnosis and treatment of diabetes mellitus and its complications. Theranostic approaches have been used in controlling insulin delivery in diabetic subjects [1]. Diabetes monitoring also benefits from theranostics as advanced blood glucose sensors [2]. Other types of theranostics inhibit carbohydrate digestive enzymes in the gastrointestinal tract and effectively reduce postprandial blood glucose levels [3].

In a recently published study, Kalfaoglu reported that diabetic kidney disease, a diabetic chronic complication, could be easily diagnosed by advanced sonographic methods [4]. Kalfaoglu showed that subjects with diabetic kidney disease had significantly higher right renal echointensity and left renal echointensity, while having lower splenic/renal echointensity and hepatic/renal echointensity ratios compared to the controls [4]. This tool could add a lot to the current diagnostic methods for this chronic complication of diabetes mellitus.

Another study claimed that systemic immune inflammation ratio (SII) could be a cost-effective predictor of diabetic kidney disease in patients with type 2 diabetes mellitus [5]. Taslamacioglu Duman et al. reported that SII levels of the patients with diabetic kidney disease were significantly higher than those of the controls [5]. The inexpensive and easy-to-assess nature of SII could make it a useful aid in establishing the timely diagnosis of diabetic kidney disease.

A future perspective of the theranostic approaches in diabetes mellitus may include enhancing insulin release from pancreatic beta cells. Nucleic acid-based therapy is emerging in all medical fields including diabetes mellitus. Recent evidence suggests that pancreatic beta cell dysfunction has been linked to changes in the expression of microRNAs [6]. A certain type of microRNA, microRNA-127, is expressed in human pancreatic islet cells. Downregulation of this microRNA results in improved beta cell proliferation and insulin secretion [7]. Targeting these microRNAs may have the potential to slow or stop diabetes mellitus. These oligonucleotides would be delivered to pancreatic islet cells and simultaneously monitored by theranostic approaches.

Another potential role of theranostic approaches could be enhanced drug delivery. Theranostics may be useful in delivery of oral insulin formulations [8] and monitoring blood glucose levels [2]. Ongoing studies on this subject are quite promising.

One more possible theranostic approach may be the use of theranostics in the diagnosis and treatment of diabetic complications [9]. For example, theranostic contrast media may establish the diagnosis of vascular problems in subjects with diabetic foot ulcers, while another theranostic nanoparticle may deliver a vasodilator drug to the affected site. The vasculature of the diabetic patients can be improved by theranostic approaches [10]. Therefore, theranostic studies on the diagnosis and treatment of diabetes mellitus and diabetic complications are awaited with great hope.

In summary, theranostic approaches may have the potential to shape the treatment of diabetes mellitus in the future. Hence, studies on this topic must be encouraged.
